# Awareness, Perceptions, and Use of Oral Nicotine Pouches Among Jazan University Students in Saudi Arabia: A Cross-Sectional Study

**DOI:** 10.3390/healthcare14010098

**Published:** 2025-12-31

**Authors:** Tariq Al Bahhawi, Alwalah H. Gaser, Wasayf M. Alamer, Shaima A. Hantul, Elham A. Najmi, Danah H. Bashiri, Mariah O. Hankish, Nouf M. Alnami, Mohammed A. Muaddi, Abdulwahab A. Aqeeli, Majed A. Ryani, Turki M. Dhayihi, Anwar S. Alahmar, Ahmed A. Bahri

**Affiliations:** 1 Family and Community Medicine Department, Faculty of Medicine, Jazan University, Jazan 45142, Saudi Arabia; tbahhawi@jazanu.edu.sa (T.A.B.);; 2 Faculty of Medicine, Jazan University, Jazan 45142, Saudi Arabia; 3 Department of Surgery, Faculty of Medicine, Jazan University, Jazan 45142, Saudi Arabia; 4 ERADH and Mental Health Hospital, Jazan Health Cluster, Jazan 82943, Saudi Arabia

**Keywords:** oral nicotine pouches, awareness, perceptions, tobacco use, university students, Saudi Arabia

## Abstract

**Background and Objectives:** Oral nicotine pouches (ONPs) are rapidly expanding nicotine products with limited evidence from the Middle East, particularly among young adults. This study assessed the awareness, perceptions, and use of ONPs among university students in Jazan, Saudi Arabia. **Materials and Methods:** A cross-sectional survey (November 2024–April 2025) used multistage stratified random sampling across six colleges at Jazan University. A self-administered questionnaire captured sociodemographic characteristics, tobacco-use history, ONPs awareness (aided), ever use and current use (past 30 days), and self-reported perceptions items across nine domains. Multivariable logistic regression estimated adjusted odds ratios (aORs) with 95% confidence intervals (CIs). **Results:** Among 624 students (mean age = 20.9 ± 1.7 years; 50.5% female), ONPs awareness was 69.7%, ever use 11.5%, and current use 7.5%. Awareness and use were higher among males and other tobacco users (*p* < 0.001). In multivariable models, male sex predicted awareness, ever use, and current use; rural residence was linked to lower awareness (aOR = 0.67; 95% CI 0.45–0.98), and being a medical student was linked to lower current use (aOR = 0.08; 95% CI 0.003–0.51) Most students perceived ONPs as addictive (80%) and harmful (68%), yet accessible (61%) and attractive (55%). **Conclusions:** ONPs awareness and use were high, particularly among males and tobacco users. Despite recognizing potential harm, students viewed ONPs as accessible and attractive. Ongoing surveillance, education, and balanced regulation are needed to guide harm-reduction policy and prevent unintended nicotine uptake.

## 1. Introduction

Tobacco use remains one of the leading preventable causes of morbidity and mortality worldwide. In 2019, smoking was responsible for about 7.7 million deaths worldwide and 200 million disability-adjusted life years [1]. It was the top cause of death among men, accounting for roughly one in five male deaths [1]. Although cigarette smoking has declined in many countries [1], the tobacco and nicotine industry has diversified by introducing novel products marketed as safer or more convenient alternatives to smoking [2,3].

A recent innovation from the tobacco industry is oral nicotine pouches (ONPs), which are tobacco-free products composed of nicotine, flavorings, and other additives [2,3]. These pouches are placed between the lip and gum, where their contents dissolve and are absorbed directly into the bloodstream without combustion [2,3]. While ONPs eliminate combustion-related toxicants found in cigarette smoke, nicotine itself poses significant biological risks. Nicotine is a highly addictive psychoactive alkaloid that activates nicotinic acetylcholine receptors, triggering dopamine release and dependence [4,5]. It also causes cardiovascular effects including increased heart rate and blood pressure [6,7]. In young adults, nicotine can disrupt brain development, impair cognitive function, and increase addiction susceptibility [5,8]. These nicotine-specific harms underscore that ONPs are not risk-free alternatives, particularly among university students.

ONPs have rapidly gained popularity, with monthly sales in the US increasing from 327 million in July 2021 to over one billion in May 2024 [9]. In the United Kingdom, the prevalence of adult ever use and current use doubled between 2020 and 2024 [10]. In parallel, concerns have emerged regarding their appeal to youth, patterns of dual or poly-use with other nicotine products, and widespread misperceptions of risk [10,11]. In Europe and North America, patterns of ONP use demonstrate consistently higher adoption among males, cigarette smokers, and e-cigarette users [12,13].

Despite growing international attention, evidence from Saudi Arabia remains limited and methodologically constrained. Existing estimates suggest high ONP use, with prevalence ranging from 14.2% [14] to 21.2% [15] in convenience samples recruited online. While informative, these studies have limited representativeness and generalizability. In addition, data on university students are scarce, even though this group often adopts novel nicotine products early and may influence future population patterns. To address these gaps, we conducted a probability-based survey of Jazan University students to (1) estimate ONP awareness and perceptions, (2) assess the prevalence of ONP use, and (3) identify demographic and behavioral factors of ONP use.

## 2. Materials and Methods

### 2.1. Design, Setting and Population

A cross-sectional study was conducted between November 2024 and April 2025 at Jazan University, the only university in the Jazan region of Saudi Arabia. The university enrolls more than 56,000 students across twenty-three colleges. The study population included all undergraduate students registered for the academic year 2024–2025.

### 2.2. Sampling Technique

A multi-stage stratified random sampling technique was applied. In the first stage, all colleges were stratified into three categories: health-related, scientific, and humanities/arts. In the second stage, two colleges were randomly selected from each category: Medicine and Nursing (health-related), Sciences and Engineering (scientific), and Business and Law (humanities/arts). In the final stage, intact classes were randomly selected within each college until the target sample size was achieved, enrolling approximately forty male and forty female students per college.

### 2.3. Sample Size

The minimum required sample size was estimated using the single-proportion formula, assuming a 95% confidence level (Z = 1.96), a proportion (*p*) of 0.5 to maximize variability, and a margin of error (d) of 0.05. The initial estimate of 384 participants was adjusted for the multi-stage clustered design using a design effect of 1.5, yielding a target of 576 participants. To account for potential non-response, an additional 10% was added, resulting in a target of 630 students. A total of 640 questionnaires were distributed, and 624 valid responses were obtained, representing a response rate of 97.5%.

### 2.4. Measures

The primary outcomes were awareness, perception, and use of ONPs. In the first page of the questionnaire there was an image and a brief description of ONPs to ensure accurate identification. Awareness was assessed using the question: “Before today, have you ever heard of nicotine pouches?” (response options: Yes/No). Perceptions were measured through nine statements regarding ONPs harm, addictiveness, affordability, attractiveness, accessibility, social acceptability, smoking cessation, smoking initiation, and comparison with nicotine-replacement therapies. The perception domains were informed by constructs used in previous studies on tobacco and ONPs and were adapted to the local context [16,17]. Each item was rated on a 5-point Likert scale (1 = strongly disagree to 5 = strongly agree). Ever use was measured by asking: “Have you ever used nicotine pouches in your lifetime?” (Yes/No). Current use was defined as use on one or more days in the past 30 days, based on the question: “During the past 30 days, on how many days did you use a nicotine pouch?”. Use of other tobacco products (cigarettes, shisha, and e-cigarettes) was assessed for both lifetime and current use, using items adapted from the Arabic version of the WHO Tobacco Questions for Surveys (TQS-Youth) [18]. Sociodemographic variables included age, sex, residence, income, and college.

### 2.5. Data Collection Method

Data were collected using a structured, Arabic, self-administered paper-based questionnaire composed of three sections: (1) sociodemographic characteristics, (2) tobacco use history, and (3) ONP awareness, perception, and use. A pilot study was conducted among twenty-five students to evaluate clarity, relevance, and comprehension of items. Minor wording adjustments were made based on feedback to enhance clarity, with no substantive changes to item content or structure. Content validity was assessed through review by the research team with expertise in public health and tobacco control, and internal consistency for perception items demonstrated acceptable reliability (Cronbach’s α = 0.85). Completed questionnaires were entered into Google Forms, exported to Microsoft Excel. Data were cleaned prior to analysis by screening for completeness and internal consistency, excluding responses with substantial missing data, and recoding variables according to predefined coding schemes.

### 2.6. Data Analysis

All analyses were performed using R software (version 4.1). Descriptive statistics were computed to summarize participant characteristics. Categorical variables were presented as frequencies and percentages, whereas continuous variables were summarized as means and standard deviations. Group differences were assessed using the Chi-square test or Fisher’s exact test for categorical variables and the independent sample *t*-test for continuous variables. Perceptions of ONPs were analyzed using the “likert” package in R [19], which allowed construction of descriptive Likert-type summaries and visualization of agreement distributions across perception domains. Each perception item was presented as a proportion of respondents who agreed, disagreed, or were neutral toward the statement. Associations between sociodemographic or behavioral factors and nicotine pouch awareness or ever use were examined using multivariable logistic regression models including sex, age, residence, income, and ever use of cigarettes, shisha, and e-cigarettes. Adjusted results were reported as odds ratios (ORs) with corresponding 95% confidence intervals (95% CIs). Statistical significance was defined as *p* < 0.05 (two-tailed). Missing data were minimal and managed using complete case analysis.

### 2.7. Sensitivity Analysis

A sensitivity analysis was conducted to explore poly-tobacco use patterns as a secondary, exploratory analysis to generate hypotheses for future research. Participants were grouped into mutually exclusive categories based on their reported use of other nicotine products used (cigarettes, shisha, and e-cigarettes) derived from separate yes/no questions for each product. These categories represent use patterns at the time of survey completion rather than stable user classifications. For each group, the prevalence of ONPs ever use and current use was calculated and compared descriptively.

### 2.8. Ethical Considerations

Ethical approval was obtained from the Jazan University Standing Committee for Scientific Research (Reference No.: REC-46/06/1279, dated 24 December 2024). Participation was voluntary, and written informed consent was obtained from all respondents.

## 3. Results

A total of 624 students participated, with a mean age of 20.9 years (SD 1.7). The sample was balanced by sex (50.5% female), and slightly more than half resided in rural area (52.1%). Approximately one-third reported a household income above SAR 15,000 per month. Tobacco use was widespread: nearly one in five students reported ever using cigarettes, e-cigarettes, or shisha, and 6–8% reported current use of these products (Table 1).

As shown in Table 2, awareness of ONPs was high (69.7%) and significantly greater among males, older students, and those with prior use of cigarettes, shisha, or e-cigarettes (all *p* < 0.001). Ever use (11.5%) followed a similar pattern, with additional significant differences across income levels (*p* = 0.018). Current use (7.5%) also differed significantly by sex, college field, income, and prior tobacco use (*p* < 0.05). Sensitivity analysis of poly-tobacco patterns showed a dose–response relationship between the number of other nicotine products used and ONPs uptake. ONPs ever use was low among non-users of cigarettes, shisha, and e-cigarettes (1.8%) but increased sharply among dual-product users (22.7–40.9%) and was highest among triple-product users (52.7%). A similar pattern was observed for current ONPs use, ranging from 1.1% in non-users to 35.1% among triple users (Table S1).

In multivariable logistic regression (Table 3), male sex and prior e-cigarette use remained independent predictors of ONPs awareness, while residence became significant and associations with age, cigarette use, and shisha use were no longer significant. For ONPs ever use, male sex and prior e-cigarette and shisha use remained significant, whereas the effects of age, cigarette use, and income observed in bivariate analyses were attenuated. For current ONPs use, male sex and prior shisha use remained significant predictors, and being enrolled in medicine showed a protective association, while income, age, e-cigarette, and cigarette use were no longer significant after adjustment.

**Figure 1:**
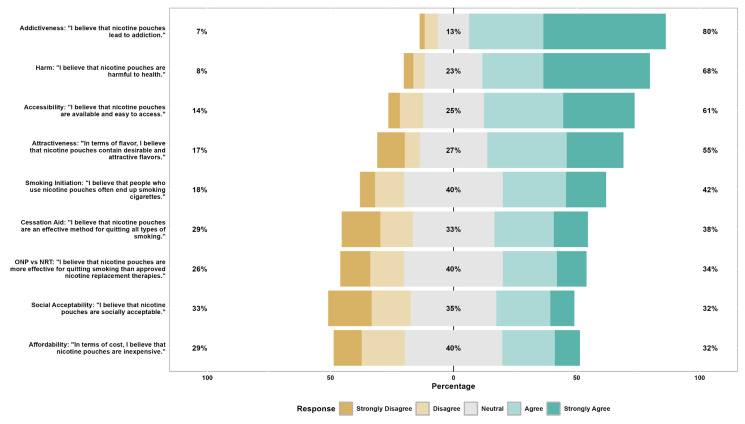
Overall perception of nicotine pouch characteristics among participants (n = 624). Stacked bars show the distribution of responses on a 5-point Likert scale for nine items: addictiveness–harm–accessibility–attractiveness/flavors–smoking initiation–cessation aid–ONP versus NRT effectiveness–social acceptability–and affordability. Right-hand percentages denote the proportion agreeing or strongly agreeing with each statement. Left-hand percentages denote the proportion disagreeing or strongly disagreeing. ONP = oral nicotine pouch; NRT = nicotine replacement therapy.

Overall perceptions of ONPs showed substantial variation across domains (Figure 1). Most students perceived ONPs as addictive (80%) and potentially harmful to health (68%), with more than half believing they are accessible (61%) and attractive (55%). Approximately one-third considered ONPs to be affordable, socially acceptable, and effective methods for smoking cessation.

## 4. Discussion

This study examined awareness, perceptions, and use of ONPs among Saudi university students through a representative cross-sectional survey. Our findings revealed that ONPs awareness was high (69.7%) among Jazan University students, with ever use reported by 11.5% and current use by 7.5% of participants. Males demonstrated significantly higher rates of both awareness and use compared to females, and ONPs use was strongly associated with other tobacco product use, suggesting patterns of poly-use. Notably, while most students perceived ONPs as addictive and harmful, they simultaneously viewed them as accessible and attractive, indicating complex and potentially contradictory perceptions that may influence future adoption patterns.

The high awareness (69.7%) observed in this study is consistent with recent Saudi findings among medical and general adult populations [14,20,21]. Internationally, comparable awareness levels have been reported in Australia [22] and the United Kingdom [23] but remain lower in earlier U.S. data [24]. Alongside high awareness, the prevalence of ever use (11.5%) and current use (7.5%) indicate notable experimentation and consumption rates exceeding most Western adult estimates, where current use rarely reach 4% [10,12,25], and are comparable to young-adult prevalence in Australia [22]. The rapid growth in both awareness and use may also reflect increased product availability, targeted digital promotion, and the normalization of nicotine alternatives in social media.

Consistent with previous studies, male sex [17,23] and previous tobacco use [15,26] were independent predictors of ONPs use. Small age differences were observed in bivariate analyses, but these did not remain significant after adjustment. Given the narrow age range of the sample, this likely reflects academic seniority and accumulated exposure to ONPs within the university environment rather than true age-related effects [27]. Lower awareness of ONPs among students residing in rural areas suggest limited market penetration and reduced marketing exposure compared with urban areas. The lower odds of current ONP use among medical students suggest greater awareness of health risks or stronger adherence to professional health norms discouraging nicotine use. Exploratory analysis of poly-tobacco patterns showed that ONP adoption was concentrated among individuals who used multiple nicotine products. ONP use was rare among non-users of other products (≈2%) but increased among dual- and triple-product users. Although these subgroup numbers were small, the trend is consistent with previous evidence indicating that ONPs are typically adopted as part of poly-nicotine behaviors rather than as stand-alone substitutes [17].

The perceptions observed in this study align with previous evidence showing that ONPs are widely viewed as addictive and potentially harmful yet increasingly considered as accessible and socially acceptable [16,17,28]. Collectively, these perception patterns illustrate a complex risk communication challenge. Without targeted educational and regulatory interventions, these mixed perceptions may facilitate dual use and increase nicotine dependence [13]. Clear communication strategies emphasizing the addictive potential and uncertain long-term safety profile of ONPs are warranted.

The high awareness and non-negligible prevalence of ONP use among Saudi university students indicate that ONPs have entered an early diffusion stage in Saudi Arabia. While the promotion of lower-risk nicotine products may align with public-health harm-reduction goals, current evidence remains insufficient to determine whether ONPs reduce tobacco-related harm or sustain nicotine dependence through dual use [29]. Moreover, ONPs may attract non-smokers and youth, potentially leading to nicotine initiation rather than harm reduction [30]. Independent evaluation of ONP health outcomes including risks and benefits, addiction potential, and cessation efficacy is needed before broader adoption can be considered.

These findings highlight the need for a balanced approach that supports harm-reduction objectives while preventing unintended expansion of nicotine use. Clear regulatory definitions within tobacco-control legislation, including standards for product content and labeling nicotine concentration, packaging, flavors restrictions and age limit, would help ensure responsible marketing and distribution [31]. Public education initiatives are also needed to improve understanding of ONPs risks and uncertainties regarding their long-term health effects. Finally, integrating ONPs indicators into national surveillance systems would enable continuous monitoring of prevalence, dual use, and health outcomes, providing the evidence base necessary to guide future harm-reduction policies in line with Saudi Vision 2030.

These findings have important implications for public health practice and research in Saudi Arabia. Public health authorities could integrate ONPs surveillance into existing tobacco monitoring systems to support evidence-based policy development. Healthcare providers and university health services may benefit from incorporating ONPs screening in routine assessments, given high awareness and use rates among students. Educational initiatives could provide balanced, evidence-based information acknowledging both potential risks and benefits. Future research priorities include longitudinal studies examining use trajectories, frequency patterns, and motivations for initiation and cessation. Understanding transitions between nicotine products and poly-use patterns would inform comprehensive tobacco control strategies aligned with Vision 2030’s health objectives [32]. Multi-site studies across Saudi universities could enhance national understanding and guide targeted interventions supporting the Kingdom’s commitment to population health improvement.

### Strengths and Limitations

This study employed multistage stratified random sampling across six colleges, enhancing representativeness and generalizability to the university population. The use of aided awareness with visual prompts likely improved ONP identification accuracy. The comprehensive assessment of awareness, perceptions, and use patterns provided a holistic view of ONP engagement among students. Including multiple tobacco products also enabled exploration of poly-use patterns.

However, the cross-sectional design limits the ability to examine temporal relationships and does not allow assessment of changes in nicotine product use over time, including switching, substitution, or cessation-related behaviors. The questionnaire did not include items on duration of use, motivations for use, quit attempts, or transitions between products; therefore, the study does not assume stable or exclusive (“pure”) user groups, and some degree of switching across categories cannot be excluded. Although ONPs are sometimes discussed as potential cessation or harm-reduction tools, the present study was not designed to evaluate cessation pathways, particularly in the context of concurrent use with products such as e-cigarettes. Longitudinal studies are needed to better characterize nicotine use trajectories and cessation-related behaviors.

Self-reported tobacco use may be subject to social desirability bias. Although participants were sampled within classroom clusters, analyses were conducted at the individual level. Residual within-classroom correlation cannot be excluded; however, given the exploratory nature of the study and relatively small cluster sizes, such clustering effects are unlikely to substantially influence the results. The sample was restricted to one university in southern Saudi Arabia and therefore may not be generalizable to students in other regions or institutions.

## 5. Conclusions

Among Saudi university students, ONP awareness and use were high, with clear differentials by sex and tobacco-use history. The coexistence of risk perceptions (addictive, harmful) alongside appeal factors (accessible, attractive) highlights a complex attitudinal landscape that may encourage experimentation despite health concerns. These findings provide essential baseline data to inform Saudi Arabia’s emerging tobacco-control efforts and align with Vision 2030 priorities for evidence-based public health policy. Sustained monitoring, particularly among youth, will be critical to guide balanced strategies that support harm reduction while preventing unintended expansion of nicotine use.

## Figures and Tables

**Table 1:** Sociodemographic and tobacco use characteristics of participants (n = 624).

Characteristic	n (%)
Age in years (Mean ± SD)	20.9 ± 1.7
Sex	
Female	315 (50.5)
Male	309 (49.5)
Residence	
Urban	275 (44.1)
Rural	325 (52.1)
Missing	24 (3.8)
Monthly family income (SAR)	
<5000	71 (11.4)
5000–<10,000	178 (28.5)
10,000–<15,000	149 (23.9)
≥15,000	211 (33.8)
Missing	15 (2.4)
College	
Business	108 (17.3)
Engineering and Computer Science	104 (16.7)
Medicine	104 (16.7)
Nursing and Health Sciences	101 (16.2)
Sciences	106 (17.0)
Sharia and Law	101 (16.2)
Ever tobacco use	
Cigarettes	123 (19.7)
Shisha	138 (22.1)
E-cigarettes	123 (19.7)
Current tobacco use	
Cigarettes	38 (6.1)
Shisha	48 (7.7)
E-cigarettes	42 (6.7)

n: Frequency; %: Percentage; SD: Standard deviation; SAR: Saudi riyals. Current tobacco use defined as use on ≥1 day in the past 30 days.

**Table 2:** Awareness, ever use, and current use of oral nicotine pouches by demographic characteristics (n = 624).

Characteristic	Awareness of ONPs			Ever Use of ONPs			Current Use of ONPs		
	No n (%)	Yes n (%)	*p*-Value	No n (%)	Yes n (%)	*p*-Value	No n (%)	Yes n (%)	*p*-Value
Overall	189 (30.3)	435 (69.7)	-	552 (88.5)	72 (11.5)	-	577 (92.5)	47 (7.5)	-
Age in years (Mean ± SD)	20.6 ± 1.5	21.0 ± 1.7	0.003	20.8 ± 1.6	21.6 ± 2.1	0.003	20.8 ± 1.6	21.4 ± 2.0	0.084
Sex			<0.001			<0.001			<0.001
Female	131 (41.6)	184 (58.4)		309 (98.1)	6 (1.9)		313 (99.4)	2 (0.6)	
Male	58 (18.8)	251 (81.2)		243 (78.6)	66 (21.4)		264 (85.4)	45(14.6)	
Residence			0.062			0.500			0.800
City	74 (26.9)	201 (73.1)		246 (89.5)	29 (10.5)		253 (92.0)	22 (8.0)	
Village	111 (34.2)	214 (65.8)		285 (87.7)	40 (12.3)		302 (92.9)	23 (7.1)	
Monthly family income (SAR)			0.200			0.018			0.039
<5000	20 (28.2)	51 (71.8)		59 (83.1)	12 (16.9)		63. (88.7)	8 (11.3)	
5000–<10,000	63 (35.4)	115 (64.6)		152 (85.4)	26 (14.6)		164 (92.1)	14 (7.9)	
10,000–<15,000	50 (33.6)	99 (66.4)		141 (94.6)	8 (5.4)		145 (97.3)	4 (2.7)	
≥15,000	55 (26.1)	156 (73.9)		187 (88.6)	24 (11.4)		192 (91.0)	19 (9.0)	
College			0.900			0.061			0.011
Business	31 (28.7)	77 (71.3)		89 (82.4)	19 (17.6)		92 (85.2)	16 (14.8)	
Engineering	34 (32.7)	70 (67.3)		88 (84.6)	16 (15.4)		96 (92.3)	8 (7.7)	
Medicine	30 (28.8)	74 (71.2)		99 (95.2)	5 (4.8)		103 (99.0)	1 (1.0)	
Nursing	27 (26.7)	74 (73.3)		91 (90.1)	10 (9.9)		93 (92.1)	8 (7.9)	
Sciences	33 (31.1)	73 (68.9)		94 (88.7)	12 (11.3)		99 (93.4)	7 (6.6)	
Sharia and Law	34 (33.7)	67 (66.3)		91 (90.1)	10 (9.9)		94 (93.1)	7 (6.9)	
Ever use of cigarettes	11 (8.9)	112 (91.1)	<0.001	70 (56.9)	53 (43.1)	<0.001	87 (70.7)	36 (29.3)	<0.001
Ever use of shisha	13 (9.4)	125 (90.6)	<0.001	80 (58.0)	58 (42.0)	<0.001	98 (71.0)	40 (29.0)	<0.001
Ever use of e-cigarettes	10 (8.1)	113 (91.9)	<0.001	74 (60.2)	49 (39.8)	<0.001	92 (74.8)	31 (25.2)	<0.001

*p*-values from *t*-test– Pearson’s χ^2^– or Fisher’s exact test as appropriate. ONPs: Oral nicotine pouches; SAR: Saudi riyal; SD: Standard deviation. Current use defined as use on ≥1 day in the past 30 days.

**Table 3:** Multivariable logistic regression for the association between demographic and awareness– ever use– and current use of oral nicotine pouches (n = 624).

Predictor	Awareness of ONPs			Ever Use of ONPs			Current Use of ONPs		
	aOR	*p*-Value	95% CI	aOR	*p*-Value	95% CI	aOR	*p*-Value	95% CI
Age in years	1.13	0.071	0.99–1.29	1.13	0.2	0.92–1.39	1.09	0.5	0.85–1.40
Sex (Ref.: Female)									
Male	2.18	<0.001	1.46–3.26	8.96	<0.001	3.64–25.84	15.2	<0.001	4.16–98.22
College (Ref.: Business)									
Engineering	1.04	0.90	0.54–2.00	1.74	0.3	0.60–5.10	0.66	0.5	0.19–2.12
Medicine	1.42	0.3	0.71–2.82	0.37	0.2	0.07–1.49	0.08	0.026	0.003–0.51
Nursing	1.46	0.3	0.74–2.90	0.94	0.90	0.29–2.93	0.96	0.90	0.27–3.17
Sciences	1.17	0.6	0.60–2.26	1.12	0.8	0.37–3.31	0.69	0.5	0.19–2.28
Sharia And Law	1.16	0.7	0.60–2.22	1.07	0.90	0.34–3.22	0.84	0.8	0.24–2.74
Residence (Ref.: Urban)									
Rural	0.67	0.043	0.45–0.98	1.30	0.5	0.64–2.66	0.87	0.7	0.39–1.91
Monthly family income (SAR) (Ref.: <5000)									
<5000	0.86	0.6	0.43–1.64	0.97	0.90	0.34–2.81	0.75	0.6	0.23–2.46
5000–<10,000	1.04	0.90	0.51–2.04	0.36	0.11	0.10–1.23	0.33	0.13	0.07–1.37
10,000–<15,000	1.31	0.4	0.66–2.52	0.93	0.9	0.32–2.74	1.32	0.6	0.42–4.40
Ever use of cigarettes (Ref.: No)									
Yes	1.68	0.2	0.74–3.98	1.87	0.2	0.77–4.43	1.98	0.2	0.69–5.84
Ever use of shisha (Ref.: No)									
Yes	1.82	0.14	0.83–4.15	6.36	<0.001	2.67–15.74	8.57	<0.001	2.82–28.28
Ever use of e-cigarettes (Ref.: No)									
Yes	2.71	0.024	1.17–6.66	2.94	0.011	1.28–6.79	1.19	0.7	0.44–3.11

ONPs: oral nicotine pouches; aOR: adjusted Odd Ratio; CI: Confidence Interval; SAR: Saudi riyal. Current use defined as use on ≥1 day in the past 30 days.

## Data Availability

The data are not publicly available due to ethical and institutional restrictions. The dataset is held by the Department of Family and Community Medicine. Data may be made available by the authors upon reasonable request and with departmental approval.

## Supplementary Materials

The following supporting information can be downloaded at: https://www.mdpi.com/article/10.3390/healthcare14010098/s1, Table S1: Poly-use of Cigarettes, Shisha, and E-cigarettes with Nicotine Pouch (ONPs) Use.

## Abbreviations

The following abbreviations are used in this manuscript:

ONPs	oral nicotine pouches
SAR	Saudi Arabian Riyal
aOR	adjusted odds ratio
CI	confidence interval
