# Supplementary material for: Awareness, Perceptions, and Use of Oral Nicotine Pouches Among Jazan University Students in Saudi Arabia: A Cross-Sectional Study
Source: Healthcare (Basel). 2025 Dec 31;14(1):98. doi: 10.3390/healthcare14010098 (PMC12785595; doi:10.3390/healthcare14010098)
Supplement: Supplementary file 1 [file healthcare-14-00098-s001.zip › healthcare-4032306-supplementary.pdf]

Table S1. Poly-use of cigarettes, shisha, and e-cigarettes with nicotine pouch (ONPs) use

PolyGroup	*N*	*ONPs Ever* *n (%)*	*ONPs Current Use* *n (%)*
**Non-users**	442	8 (1.8)	5 (1.1)
**Cigarettes + Shisha + E-Cigarettes**	74	39 (52.7)	26 (35.1)
**Cigarettes + Shisha**	22	9 (40.9)	8 (36.4)
**Shisha**	22	5 (22.7)	3 (13.6)
**Shisha + E-Cigarettes**	20	5 (25%)	3 (15)
**E. Cigarettes**	17	1 (5.9)	0 (0)
**Cigarettes**	15	1 (6.7)	0 (0)
**Cigarettes + E-Cigarettes**	12	4 (33.3)	2 (16.7)
